# Development of an Integrated UPLC-QTOF-MS/MS and HPLC-QqQ-MS/MS Method for Polyphenol Analysis in Sea Buckthorn Juice

**DOI:** 10.3390/foods15213773

**Published:** 2026-10-22

**Authors:** Xiaoxiao Chen, Jinlei Zhang, Lu Bai, Xia Chen, Fei Zhang, Yongfa Chen, Jingfeng Zhou, Yanyan Hu, Rui Zhang, Shiyue Liu, Siqi Li, Lijie Xing, Yuan Wang

**Affiliations:** 1 Analysis and Testing Center, Xinjiang Academy of Agricultural and Reclamation Science, Shihezi 832000, China; 2 Instrumental Analysis Center, Xi’an Jiaotong University, Xi’an 710115, China

**Keywords:** sea buckthorn juice, polyphenols, UPLC-QTOF-MS/MS, HPLC-QqQ-MS/MS, screening, quantification

## Abstract

An integrated analytical method combining ultra-performance liquid chromatography coupled to quadrupole time-of-flight tandem mass spectrometry (UPLC-QTOF-MS/MS) for qualitative screening and high-performance liquid chromatography coupled to triple-quadrupole tandem mass spectrometry (HPLC-QqQ-MS/MS) for targeted quantification was developed for the comprehensive analysis of polyphenols in sea buckthorn juice. UPLC-QTOF-MS/MS screening yielded 42 candidate polyphenol annotations, from which 15 representative compounds spanning phenolic acids, flavan-3-ols, and flavonols were selected for targeted quantification. The optimized HPLC-QqQ-MS/MS conditions used acetonitrile–0.1% aqueous formic acid as the mobile-phase system, extraction with 70% ethanol followed by treatment with 0.01 mol/L HCl, and a sample-to-solvent ratio of 1:15 (g/mL). The quantitative method showed coefficients of determination (R^2^) > 0.99, LODs of 0.0010–0.0054 μg/g, LOQs of 0.0033–0.0179 μg/g, and recoveries of 76.8–105.7% with RSDs ≤ 4.4%. The established method was applied to seven sea buckthorn juice samples, with the summed concentrations of the 15 target polyphenols ranging from 34.25 to 94.86 μg/g. Sample No. 7 (Daguo Shaji No. 7) showed the highest summed concentration of the 15 target polyphenols, while rutin was the most abundant quantified compound in all seven samples. The developed method links high-resolution mass spectrometric screening with validated targeted quantification, enabling the selection of target polyphenols based on the chemical composition of actual samples and providing a reliable analytical approach for polyphenol profiling and quantification in sea buckthorn juice.

## 1. Introduction

Sea buckthorn (*Hippophae rhamnoides* L.), a perennial deciduous member of the Elaeagnaceae, is widely distributed throughout the temperate, subarctic, and alpine regions of Eurasia. As a distinctive berry resource, it is valued for both its nutritional quality and its potential for food processing and utilization [1,2,3]. In recent years, increasing attention to naturally derived food bioactives and plant-based antioxidant resources has further highlighted the value of sea buckthorn. The berries are rich in vitamins, organic acids, carotenoids, fatty acids, phytosterols, polysaccharides, polyphenols, and other nutritional and phytochemical constituents. This diverse composition makes sea buckthorn a promising raw material for the development of juices, fruit powders, fermented products, functional foods, and natural antioxidant ingredients [4,5].

Polyphenols represent an important group of secondary metabolites in sea buckthorn and mainly comprise phenolic acids, flavonols and their glycosides, flavan-3-ols, and proanthocyanidins [6,7,8]. Beyond their contribution to antioxidant properties, these compounds are closely associated with several sensory and physicochemical attributes of the fruit and its processed products, including astringency, bitterness, and oxidative stability [9,10]. However, the chemical characteristics of sea buckthorn are not solely determined by its total phenolic or total flavonoid contents, but are also closely associated with the types, concentrations, and relative proportions of individual polyphenolic compounds [10,11]. Therefore, reliable qualitative and quantitative analysis of individual polyphenols in sea buckthorn is important for characterizing its polyphenol composition.

Current approaches for the analysis of sea buckthorn polyphenols mainly include colorimetric assays for total phenolics or total flavonoids, high-performance liquid chromatography coupled with diode-array and ultraviolet detection (HPLC-DAD/UV), and liquid chromatography–mass spectrometry (LC–MS). Colorimetric methods are simple and convenient for estimating the overall level of phenolic compounds in a sample, but they are susceptible to interference from reducing sugars, organic acids, vitamin C, and other co-extracted constituents. More importantly, they provide no information on individual compounds [12,13]. HPLC-DAD/UV can separate and quantify certain known phenolic acids and flavonoids, yet compound identification relies primarily on retention times and UV absorption characteristics. When the sample matrix is complex, particularly in the presence of isomers or co-elution, compound identification can be limited, and structural information such as accurate molecular masses and fragment ions is difficult to obtain [14,15].

High-resolution LC–MS, particularly UPLC-QTOF-MS/MS, is well suited for broad screening as well as structural annotation of polyphenolic compounds in complex food matrices [16,17]. The combined use of accurate-mass measurements, MS/MS fragmentation data, and database matching can substantially improve the reliability of candidate compound identification. This strategy has been successfully applied to the systematic profiling of polyphenols and other bioactive constituents in sea buckthorn [18,19]. However, QTOF-MS/MS is mainly used for non-targeted or suspect screening, and its sensitivity, selectivity, and quantitative repeatability in the routine quantification of low-abundance compounds and the analysis of large sample batches may still be affected by complex matrices.

In contrast, triple–quadrupole tandem mass spectrometry coupled with multiple reaction monitoring (MRM) offers high selectivity and sensitivity, while effectively minimizing interference from co-eluting components in complex matrices. It is therefore well suited to the targeted quantification of multiple polyphenolic compounds [20,21]. Wei et al. [22] employed LC-MS/MS to systematically analyze a broad range of phenolic acids, flavonoids, and their glycosides in sea buckthorn, enabling the quantitative determination of multiple polyphenolic compounds. Wang et al. [23] established an HPLC-MS/MS approach for simultaneously quantifying 32 polyphenolic compounds in berry samples and successfully applied the method to the analysis of sea buckthorn, mulberry, goji berry, and other berry samples. These studies indicate that this technique can meet the quantitative requirements for multiple target compounds in complex berry matrices. However, HPLC-QqQ-MS/MS used as a stand-alone technique generally requires predefined target compounds and relies on authentic standards for the selection of MRM transitions and construction of calibration curves, making it difficult to cover polyphenolic compounds outside the predefined target list, particularly unknown compounds.

Accordingly, using sea buckthorn berries as the starting material and the resulting juice as the analytical matrix, the present study aimed to establish an analytical method integrating UPLC-QTOF-MS/MS-based qualitative screening with HPLC-QqQ-MS/MS-based targeted quantification for the analysis of polyphenol compositional characteristics in sea buckthorn juice. First, UPLC-QTOF-MS/MS was employed for broad screening and preliminary structural annotation of candidate polyphenolic compounds in the samples, providing qualitative information to guide target compound selection. Subsequently, representative compounds covering phenolic acids, flavan-3-ols, and flavonols were selected to develop an HPLC-QqQ-MS/MS method for targeted quantification. The two mass spectrometric techniques served complementary analytical purposes: UPLC-QTOF-MS/MS provided broader information on candidate polyphenol composition and assisted in target selection, whereas HPLC-QqQ-MS/MS enabled selective quantification of the selected polyphenolic compounds. Finally, the established quantitative method was applied to seven large-fruited sea buckthorn juice samples to evaluate its applicability to real-sample analysis and provide quantitative information on individual target polyphenols, thereby supporting the characterization of polyphenol composition in sea buckthorn juice.

## 2. Materials and Methods

### 2.1. Materials, Reagents, and Instruments

#### 2.1.1. Samples

Fresh sea buckthorn berries were provided by Xinjiang 170th Regiment Silk Road Sea Buckthorn Biotechnology Co., Ltd. and collected from the 170th Regiment, Emin County, Tacheng Prefecture, Xinjiang, China (46°18′46″ N, 84°39′47″ E; altitude: 684.4 m). The berries were harvested in 2025 during the first picking of early-maturing sea buckthorn. Seven sea buckthorn samples were included, designated as Daguo Shaji No. 1 through No. 7. After collection, damaged berries, leaves, branches, and other impurities were removed. The samples were individually packed in sealed resealable bags, labeled, and stored at −20 °C in the sample storage room of the Xinjiang Academy of Agricultural and Reclamation Science.

#### 2.1.2. Reagents and Standards

Acetonitrile, methanol, and formic acid of HPLC grade, together with absolute ethanol and ammonium acetate of analytical grade, were obtained from Anpel Laboratory Technologies Inc. (Shanghai, China). Methanol of analytical grade was obtained from Sinopharm Chemical Reagent Co., Ltd. (Shanghai, China). Analytical-grade ethyl acetate was supplied by Tianjin Fuyu Fine Chemical Co., Ltd. (Tianjin, China). Poly-Sery HLB Pro cartridges for solid-phase extraction (SPE; 500 mg/6 mL) and membrane filters with a pore size of 0.22 μm were obtained from Anpel Laboratory Technologies Inc.

Reference standards for the 15 target polyphenols were obtained from Anpel Laboratory Technologies Inc., including gallic acid, (−)-epigallocatechin, protocatechuic acid, p-hydroxybenzoic acid, (+)-catechin, neochlorogenic acid, p-coumaric acid, caffeic acid, ferulic acid, (−)-epicatechin, vanillic acid, rutin, salicylic acid, quercetin, and isorhamnetin.

#### 2.1.3. Instruments and Equipment

High-resolution MS measurements were acquired with a Vion IMS QTof LC–QTOF-MS platform (Waters, Milford, MA, USA). Targeted quantitative analysis was conducted using an Agilent 1200 HPLC coupled with a model 6460 triple-quadrupole MS system (Agilent Technologies, Santa Clara, CA, USA). Major sample-preparation equipment included an analytical balance (Sartorius, Göttingen, Germany), vortex mixer (IKA, Staufen, Germany), ultrasonic cleaner (Kunshan Ultrasonic Instruments Co., Ltd., Kunshan, China), refrigerated centrifuge (Thermo Fisher Scientific, Waltham, MA, USA), rotary evaporator (EYELA, Tokyo, Japan), nitrogen evaporator (Organomation, Berlin, MA, USA), and ultrapure water system (Milli-Q, Burlington, MA, USA).

### 2.2. Standard Solution Preparation

Individual stock solutions of the polyphenol standards were first prepared separately in methanol and then combined to prepare a mixed intermediate standard solution. This mixture was serially diluted with methanol–water (1:1, *v*/*v*) to obtain working standards at 2.0, 5.0, 10.0, 20.0, 50.0, 100.0, 200.0, and 500.0 ng/mL. Appropriate concentration levels were selected for each target analyte according to its instrumental response and linear range to construct the calibration curves. The working standard solutions were freshly prepared before use.

### 2.3. Sample Preparation

#### 2.3.1. Sample Preparation for UPLC-QTOF-MS/MS Screening

Based on previous reports on sea buckthorn polyphenol extraction [24,25], 70% ethanol was selected for preliminary screening. Frozen sea buckthorn berries were naturally thawed, washed, and drained, and then juiced using a juice extractor–separator. The pomace was separated and removed, and the juice was collected. The collected juice was not subjected to any additional clarification, pasteurization, or other processing treatments and is hereafter referred to as the original juice sample. This original juice sample served as the analytical matrix for subsequent polyphenol extraction. Specifically, an aliquot of 2.00 g of the original juice sample (weighed to the nearest 0.01 g) was combined with 20 mL of 70% ethanol in a capped 50 mL centrifuge tube. The mixture was vortex-mixed for 1 min and ultrasonically extracted for 30 min at 50 °C. The extract was subsequently centrifuged for 5 min at 10,000 rpm. The resulting supernatant was passed through a 0.22 μm membrane filter, and the filtered extract was used as the final injection solution for UPLC-QTOF-MS/MS analysis.

#### 2.3.2. Preparation of Samples for HPLC-QqQ-MS/MS Targeted Quantification

Daguo Shaji No. 1 was used as the representative sample to assess the influence of solvent type and sample-to-solvent ratio on the extraction of target polyphenols. Ten extraction conditions were tested, including methanol, ethanol, ethyl acetate, ultrapure water, 0.01 mol/L hydrochloric acid, 70% methanol, 70% ethanol, and combined treatments of 70% methanol, 70% ethanol, or ethyl acetate with 0.01 mol/L hydrochloric acid. Five sample-to-solvent ratios, namely 1:5, 1:10, 1:15, 1:20, and 1:25 (g/mL), were examined. For the acid-assisted extraction systems, the sample-to-solvent ratio refers to the initial organic-solvent extraction.

A total of 2.00 g of the original sea buckthorn juice (weighed to the nearest 0.01 g) was combined with the corresponding extraction solvent at the designated ratio, vortexed for 1 min, ultrasonically extracted for 30 min at 50 °C, and subsequently centrifuged for 5 min at 10,000 rpm. For methanol, ethanol, ethyl acetate, ultrapure water, 0.01 mol/L hydrochloric acid, 70% methanol, and 70% ethanol, the supernatant was collected after centrifugation. For the acid-assisted extraction systems, the samples were first extracted with the corresponding organic solvent, and the organic-phase supernatant was collected and evaporated to dryness. Subsequently, 0.01 mol/L hydrochloric acid (HCl), in a volume equal to that of the organic extract, was added to the sample residue remaining after the first extraction for a second extraction. After centrifugation, the acidic supernatant was collected and used to reconstitute the dried residue obtained from the first organic solvent extraction, thereby combining the organic solvent extract and the acid-assisted extract. The combined extract was centrifuged again, and the resulting supernatant was subjected to HLB solid-phase extraction (SPE) for purification.

HLB SPE cartridges were conditioned and equilibrated sequentially using 6 mL of methanol followed by 6 mL of water. Following sample loading, the cartridges were washed with 6 mL of water, drained under suction, and subsequently eluted with 10 mL of methanol. The collected eluate was dried under a nitrogen stream at 40 °C. The residue was reconstituted to a final volume of 2 mL with methanol–water (1:1, *v*/*v*) and passed through a 0.22 μm membrane filter. The final filtrate was used for HPLC-QqQ-MS/MS analysis.

### 2.4. Chromatographic and Mass Spectrometric Conditions

#### 2.4.1. UPLC-QTOF-MS/MS Conditions

An ACQUITY UPLC HSS T3 C18 column (2.1 mm × 100 mm, 1.8 μm; Waters, Milford, MA, USA) was used for chromatographic separation. Mobile phase A was water containing 0.1% formic acid, whereas mobile phase B was acetonitrile. Gradient elution was programmed as follows: 0–0.5 min, 95% A; 0.5–10 min, 90–50% A; 10–11 min, 50–5% A; 11–13 min, 5% A; 13–14 min, 5–95% A; and 14–15 min, 95% A. The separation was carried out at a flow rate of 0.5 mL/min with the column maintained at 40 °C and an injection volume of 5 μL.

MS data were collected under negative electrospray ionization. Full-scan acquisition covered an *m*/*z* range of 50–1000. The capillary voltage was 2.50 kV, while the source and desolvation temperatures were maintained at 100 and 550 °C, respectively. Desolvation and cone gas were supplied at flow rates of 1000 and 50 L/h, respectively. Collision energy was fixed at 6 eV for the low-energy channel and ramped from 20 to 50 eV for the high-energy channel.

#### 2.4.2. HPLC-QqQ-MS/MS Conditions

An Agilent InfinityLab Poroshell 120 EC-C18 column (3.0 mm × 100 mm, 2.7 μm; Agilent Technologies, Santa Clara, CA, USA) was employed for chromatographic separation. The mobile phase comprised acetonitrile (A) and water containing 0.1% formic acid (B). Gradient elution was programmed as follows: 0–5 min, 5–10% A; 5–8 min, 10–15% A; 8–11 min, 15% A; 11–12 min, 15–18% A; 12–22 min, 18% A; 22–23 min, 18–90% A; 23–24 min, 90% A; 24–25 min, 90–5% A; and 25–28 min, 5% A. The chromatographic run was performed at 0.4 mL/min with the column maintained at 30 °C and an injection volume of 5 μL.

MS detection was performed in negative electrospray ionization mode using multiple reaction monitoring (MRM). The capillary voltage was 3.5 kV. The drying gas was maintained at 325 °C with a flow rate of 10 L/min, whereas the sheath gas was operated at 350 °C and 12 L/min. Nebulizer pressure was 45 psi. For each target analyte, the precursor ions, product ions, and collision energies were optimized through direct injection of the corresponding individual standard solution.

### 2.5. In-House Database and Candidate Compound Screening

An in-house database of sea buckthorn polyphenols was established using information from reference standards, ChemSpider, other public databases, and the relevant literature. Compound names, molecular formulas, theoretical exact masses, and literature information were recorded, and the corresponding structure files were imported into the data-processing software to assist compound matching. UPLC-QTOF-MS/MS data were processed in negative ionization mode. Candidate compounds were annotated by considering extracted ion chromatographic peaks, retention times, quasi-molecular ions, mass errors, and available fragment-ion information. A mass tolerance of ±5 ppm was applied.

### 2.6. Method Validation

Under the optimized chromatographic, mass spectrometric, and sample-preparation conditions, method validation covered linearity, sensitivity, and recovery for the 15 target polyphenols. Calibration curves were generated from the relationship between analyte concentration and peak area. Regression equations were then derived together with the corresponding correlation coefficients. The limits of detection (LOD) and quantification (LOQ) were estimated using signal-to-noise (S/N) criteria of 3 and 10, respectively. Recovery was assessed using mixed standard solutions spiked at 50, 100, and 200 μg/kg. Six replicate samples were prepared at each spiking level and processed and analyzed according to the complete sample preparation procedure. Recoveries were calculated from the measured concentrations before and after spiking, while RSDs were obtained from six replicate determinations at each spiking level. These parameters were used to evaluate method accuracy and repeatability, respectively.

### 2.7. Data Processing

UPLC-QTOF-MS/MS datasets were analyzed with UNIFI software (version 1.8.2.169) for data analysis, database matching, and the screening and annotation of candidate polyphenolic compounds, whereas HPLC-QqQ-MS/MS data were used for targeted quantification. Target-compound concentrations were obtained using the corresponding calibration curves and calculated based on the original juice sample mass, final extract volume, and applicable dilution factors. The results were expressed as μg/g of original sea buckthorn juice on a wet-weight basis. The measured densities of Daguo Shaji Nos. 1–7 were 1.057, 1.041, 1.039, 1.061, 1.075, 1.044, and 1.067 g/mL, respectively. Each sample was analyzed in triplicate, with results reported as mean ± standard deviation (SD). Values below the detection limit were designated as ND.

## 3. Results

### 3.1. Qualitative Screening and Annotation by UPLC-QTOF-MS/MS

**Figure 1:**
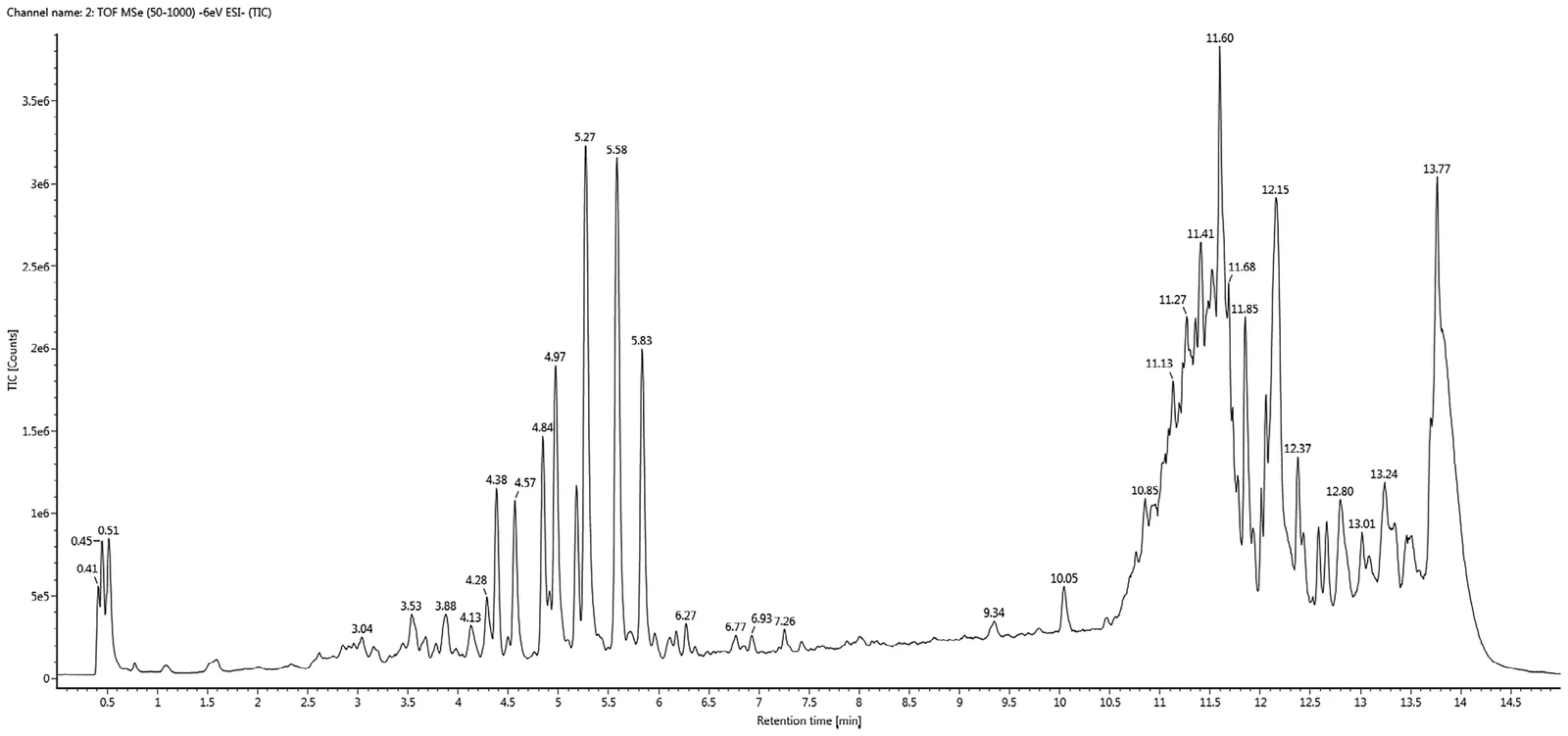
Representative total ion chromatogram (TIC) of sea buckthorn juice extract acquired by UPLC-QTOF-MS/MS in negative-ion mode.

UPLC-QTOF-MS/MS full-scan analysis was performed on sea buckthorn juice extracts in negative ionization mode, and the resulting data were screened using the polyphenol database. A representative total ion chromatogram (TIC) is shown in Figure 1. Based on the full-scan data, compound screening and structural annotation were performed by integrating extracted ion chromatograms (EICs), retention times, quasi-molecular ions, accurate-mass measurements, mass errors, and MS/MS fragmentation information. In total, 42 candidate polyphenol annotations were obtained (Table 1), with retention times ranging from 1.34 to 7.75 min and mass errors from −4.4 to 3.3 ppm, all within ±5 ppm. The candidate annotations included phenolic acids, flavan-3-ols, proanthocyanidins, flavonols and their glycosides, tannin-related compounds, stilbenes, and other flavonoids.

Among the 42 candidate polyphenols screened by UPLC-QTOF-MS/MS, 15 were selected for targeted quantification by HPLC-QqQ-MS/MS. These compounds are marked with an asterisk (*) in Table 1, and their extracted ion chromatograms obtained by UPLC-QTOF-MS/MS are shown in Figure S1.

### 3.2. Development of the HPLC-QqQ-MS/MS Targeted Quantification Method

#### 3.2.1. Selection of Target Polyphenols and Optimization of MRM Parameters

Based on the 42 candidate polyphenols screened by UPLC-QTOF-MS/MS, 15 target compounds were ultimately selected for HPLC-QqQ-MS/MS quantification by considering their representativeness in sea buckthorn, availability of reference standards, and stability of MRM responses. The selected analytes comprised nine phenolic acids, three flavan-3-ols, and three flavonols and their glycosides.

For each target analyte, an individual standard solution (1 μg/mL) was prepared and injected separately to determine suitable precursor/product ion pairs and optimize fragmentor voltages and collision energies in negative ion mode. MRM ion pairs were selected according to ion response, stability, and selectivity, and the optimized parameters are presented in Table 2. All target analytes showed stable responses in negative ESI mode and were therefore suitable for subsequent targeted quantification.

#### 3.2.2. Optimization of the Mobile Phase and Chromatographic Conditions

**Figure 2:**
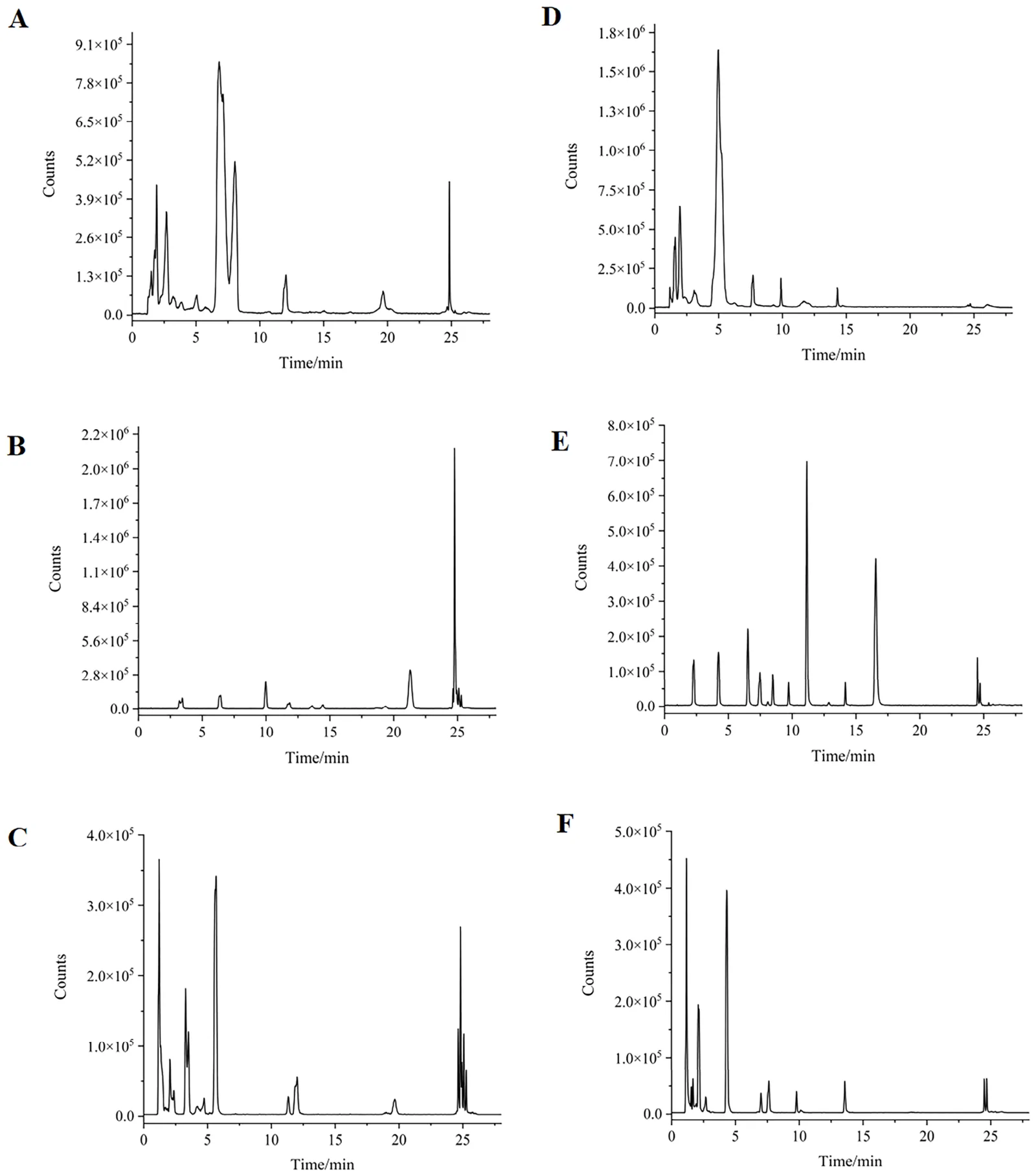
Chromatographic profiles of the target polyphenolic compounds under six mobile-phase combinations: methanol–water (**A**), methanol–0.1% aqueous formic acid (**B**), methanol–5 mmol/L ammonium acetate (**C**), acetonitrile–water (**D**), acetonitrile–0.1% aqueous formic acid (**E**), and acetonitrile–5 mmol/L ammonium acetate (**F**).

To accommodate target analytes with different structures and polarities, six binary mobile-phase combinations were evaluated, with methanol or acetonitrile paired with water, 0.1% aqueous formic acid, or 5 mmol/L ammonium acetate. Differences in peak shape, retention-time distribution, and signal response were observed among the mobile-phase systems (Figure 2).

With methanol-based systems, the retention times of some analytes were relatively clustered, and several compounds showed unsatisfactory peak shapes or responses. The acetonitrile systems containing water or 5 mmol/L ammonium acetate improved the chromatographic behavior of some analytes, but the overall chromatographic performance remained suboptimal. In contrast, the combination of acetonitrile and 0.1% aqueous formic acid produced sharper peaks for most target analytes, a more stable baseline, and a retention-time distribution better suited to multi-component MRM analysis. Therefore, this mobile-phase system was selected for subsequent quantitative analysis.

#### 3.2.3. Optimization of Sample Preparation Conditions

*Extraction system:* Daguo Shaji No. 1 was used as the representative sample to compare the extraction performance of 10 extraction systems for the target polyphenols (Table 3). Across the different extraction conditions, the total content of target compounds ranged from 2.31 to 30.75 μg/g, with 6–13 compounds detected; the complete data for individual analytes are provided in Table S1. Pure methanol and pure ethanol showed relatively weak extraction performance. After the addition of water, both the total target-compound content and the number of detected compounds increased in the 70% methanol and 70% ethanol systems.

The ethyl acetate system yielded a relatively high total target-compound content, but only 10 compounds were detected. In contrast, both water and 0.01 mol/L HCl enabled the detection of 13 target compounds, with broader extraction coverage. In the present study, sequential extraction with 70% ethanol followed by treatment of the remaining residue with 0.01 mol/L HCl increased the total target-compound content to 30.75 μg/g, approximately 8.9 times that obtained with 70% ethanol extraction alone, while the number of detected compounds increased from 9 to 13. The total target polyphenol content was 9.45% and 2.67% higher than the corresponding values obtained with the 70% methanol–0.01 mol/L HCl and ethyl acetate–0.01 mol/L HCl treatment systems, respectively. Moreover, this system gave relatively high extraction amounts of rutin, (+)-catechin, and (−)-epigallocatechin, providing both effective extraction of the major high-abundance components and broad target-compound coverage. Considering the total target-compound content, number of detected compounds, extraction performance for the major components, and solvent suitability, the 70% ethanol–0.01 mol/L HCl extraction system was selected for subsequent analysis.

*Sample-to-solvent ratio:* After selection of the extraction system, sample-to-solvent ratios from 1:5 to 1:25 were further evaluated (Table 3), and the complete data for individual analytes are provided in Table S2. As the extraction-solvent volume increased from 10 to 30 mL, the total content of the 15 target polyphenols increased from 29.68 to 32.52 μg/g, corresponding to an overall increase of 9.58%, while the number of detected compounds increased from 12 to 13. When the extraction-solvent volume was further increased to 40 and 50 mL, the total target-compound contents were 31.95 and 30.77 μg/g, respectively, both lower than that obtained with 30 mL, whereas the number of detected compounds remained at 13. Considering the total target-compound content, number of detected compounds, and solvent consumption, a sample-to-solvent ratio of 1:15 (2.00 g:30 mL) was selected for subsequent sample preparation.

#### 3.2.4. Method Validation Results

Under the optimized instrumental and sample-preparation conditions, validation of the method was performed for the 15 target polyphenols with respect to linearity, sensitivity, accuracy, and repeatability. The calibration curves were constructed from mixed standard solutions containing the 15 compounds. All analytes exhibited good linearity over their respective concentration ranges, with coefficients of determination (R^2^) > 0.99. The LODs were 0.0010–0.0054 μg/g, whereas the LOQs were 0.0033–0.0179 μg/g (Table 4), showing high analytical sensitivity.

For the spike-recovery experiments, samples were spiked at 50, 100, and 200 μg/kg, and six replicate samples were prepared for each level. Table 5 presents the recoveries and corresponding relative standard deviations (RSDs) for the 15 target polyphenols. Across the three spiking levels, the recoveries were in the range of 76.8–105.7%, and the RSDs were in the range of 0.8–4.4%. These results demonstrate that the analytical method has satisfactory accuracy and repeatability, supporting its use for quantitative analysis of the 15 target polyphenols in sea buckthorn samples.

### 3.3. Quantitative Determination of Target Polyphenols in Sea Buckthorn Juice Samples

The established method was applied to determine the 15 target polyphenols in sea buckthorn juice samples (Daguo Shaji Nos. 1–7) (Table 6), with detailed quantitative data for each compound provided in Table S3. The total contents of the target polyphenols ranged from 34.25 to 94.86 μg/g and followed the descending order: sample No. 7 > No. 5 > No. 2 > No. 3 > No. 4 > No. 6 > No. 1. The highest value was approximately 2.77 times the lowest.

Rutin was the most abundant target compound in all seven samples, with concentrations ranging from 30.05 to 72.10 μg/g and accounting for 73.72–92.96% of the total target-polyphenol content. By contrast, flavan-3-ols such as (+)-catechin, (−)-epigallocatechin, and (−)-epicatechin showed different concentration patterns across the analyzed samples.

## 4. Discussion

### 4.1. MS/MS Fragmentation Characteristics of Polyphenolic Compounds

The screening results of 42 candidate polyphenols indicated that sea buckthorn juice has a relatively rich polyphenol composition. The MS/MS fragmentation behavior of different structural classes was closely related to their molecular structures. To support the structural annotation of candidate compounds, the MS/MS fragmentation characteristics of representative compounds from different structural classes were examined.

Phenolic acids mainly underwent decarboxylation or side-chain cleavage. The [M–H]^−^ precursor ions of gallic acid, caffeic acid, and p-coumaric acid yielded fragment ions at *m*/*z* 125, 135, and 119, respectively, consistent with neutral loss of CO_2_ from the corresponding precursor ions [26,27]. Neochlorogenic acid showed a [M–H]^−^ precursor ion at *m*/*z* 353.0867, together with product ions at *m*/*z* 191, 179, and 173, exhibiting the typical fragmentation pattern of caffeoylquinic acids [28].

Flavan-3-ols exhibited similar fragmentation behavior of the core skeleton. Structural isomers such as (+)-catechin and (−)-epicatechin generally undergo comparable neutral loss and ring cleavage pathways in negative ion mode [29]. Both produced common product ions at *m*/*z* 245, 203, 151, 137, and 109, whereas additional ions at *m*/*z* 205 and at *m*/*z* 179 and 125 were observed for (+)-catechin and (−)-epicatechin, respectively. Overall, the observed fragmentation behavior was in agreement with previously reported MS/MS characteristics of flavan-3-ols [26,29]. Procyanidin B3 showed a [M–H]^−^ ion at *m*/*z* 577.1354, while the product ions at *m*/*z* 425, 407, 289, and 245 could be associated with retro-Diels-Alder (RDA) cleavage, subsequent dehydration, and quinone methide (QM) fragmentation pathways characteristic of B-type procyanidin dimers [30].

Flavonol glycosides mainly formed the corresponding aglycone ions through neutral loss of glycosyl moieties or combinations of sugar residues. Rutin showed a [M–H]^−^ ion at *m*/*z* 609.1468 and, after loss of the rutinosyl moiety, mainly generated the quercetin aglycone ion at *m*/*z* 301, which could undergo further fragmentation of the aglycone skeleton [31]. Similar fragmentation behavior was observed for other flavonol O-glycosides, with quercetin, isorhamnetin, and kaempferol glycosides producing aglycone-related ions near *m*/*z* 301, 315, and 285, respectively [26,32].

Most candidate compounds yielded MS/MS fragment information consistent with their structural characteristics. For protocatechuic acid, syringic acid, salicylic acid, and myricetin, reliable diagnostic fragment ions were not obtained. This may be related to differences in ionization and fragmentation behavior among compounds, their relative abundances in the samples, and the lack of individual optimization of collision parameters for each candidate compound under the broad screening conditions [29,33]. Compared with compounds supported by characteristic fragment ions, these four compounds had relatively lower confidence in their structural identification. Nevertheless, their experimentally measured accurate masses showed good agreement with the theoretical values in the database, with mass errors below 3 ppm, and these compounds have also been reported previously in sea buckthorn [22,34]. Therefore, they were classified as tentative annotations in the qualitative screening conducted in this study, and these candidate compounds may still provide complementary information for the overall characterization of the polyphenolic profile of sea buckthorn. Among them, protocatechuic acid and salicylic acid were further analyzed using corresponding reference standards and optimized multiple reaction monitoring (MRM) transitions in the subsequent HPLC-QqQ-MS/MS targeted analysis, providing additional support for their structural assignments.

### 4.2. Mobile-Phase Optimization

Polyphenolic compounds vary markedly in structure, polarity, and ionization characteristics; consequently, mobile-phase composition can substantially affect chromatographic retention, peak shape, and signal response [35,36]. In this study, the acetonitrile–0.1% aqueous formic acid system showed better overall chromatographic performance for multicomponent analysis than the other mobile-phase combinations tested. This may be related to differences in the elution selectivities of acetonitrile and methanol in reversed-phase chromatography [36], while acetonitrile–water systems generally have lower viscosity, which is favorable for mass transfer and peak efficiency [37]. As a volatile additive, low-concentration formic acid can adjust the acidity of the mobile phase and may also help improve the peak shape and retention behavior of some ionizable phenolic compounds [35,38]. By contrast, although the ammonium acetate system improved the chromatographic behavior of some analytes, its overall separation performance under the present conditions was not comparable to that of the acetonitrile–0.1% aqueous formic acid system. Therefore, the acetonitrile–0.1% aqueous formic acid system was selected as the mobile phase for subsequent HPLC-QqQ-MS/MS quantification.

### 4.3. Optimization of Sample Pretreatment Conditions

The extraction results showed that solvent composition markedly affected extraction efficiency and target-analyte coverage. In the present study, aqueous methanol and aqueous ethanol systems exhibited better overall extraction performance than the corresponding neat alcohol systems. This may be attributed to the addition of water altering the polarity of the solvent system, thereby facilitating the dissolution and release of polyphenolic compounds with different polarities [39,40]. This result was generally consistent with previous studies in which aqueous alcohol systems were commonly used for polyphenol extraction and condition optimization in fruits and plant-derived materials [41,42].

In addition to the alcohol–water ratio, the acidity of the extraction medium can also influence the extraction behavior of polyphenols. Previous studies have shown that adjusting the pH of the extraction medium or using acidified alcoholic solvents can enhance the extraction efficiency of polyphenolic and flavonoid compounds [43,44,45]. In the present study, the optimized extraction procedure used 70% ethanol as the primary extraction solvent, followed by extraction of the sample residue with an equal volume of 0.01 mol/L HCl. Before SPE purification, the resulting acidic supernatant was used to reconstitute the dried ethanol extract. This sequential extraction strategy was designed to combine the compounds recovered during the hydroalcoholic extraction step with those released during the subsequent acidic treatment. This approach may facilitate the recovery of polyphenolic compounds under different extraction conditions and reduce the possibility of incomplete extraction of certain polyphenols when a single extraction condition is used. Among the tested extraction conditions, the sequential extraction procedure using 70% ethanol followed by HCl showed the best performance in terms of both the total amount of extracted target analytes and detection coverage. This result suggests that, for the sample matrix and target-analyte range examined in this study, the combined adjustment of the alcohol–water ratio and acidity was favorable for achieving more comprehensive extraction. Considering that evaporation, reconstitution, and other preparation steps may result in analyte losses, spike-recovery experiments were conducted to evaluate the recovery performance and repeatability of the complete optimized sample preparation procedure. Spike recoveries ranged from 76.8% to 105.7%, with relative standard deviations (RSDs) of 0.8–4.4%, indicating acceptable recovery and repeatability under the evaluated conditions.

Further optimization of the sample-to-solvent ratio showed that increasing the solvent volume improved target-analyte extraction within a certain range, but this effect did not continue as the solvent volume increased further. When the sample-to-solvent ratio was increased from 1:5 to 1:15, the extraction level of the target polyphenols increased. However, further increases to 1:20 and 1:25 did not lead to a further increase in the total target-analyte content, while the number of detected compounds remained unchanged, suggesting that the extraction process had approached a plateau. A moderate increase in solvent volume may improve mass transfer, thereby promoting the release of the target analytes [42,46]. Once the solvent volume reaches a certain level, however, further increases provide limited additional extraction benefit while increasing solvent consumption and the burden associated with subsequent concentration and sample processing. Therefore, considering both extraction performance and solvent-use efficiency, a sample-to-solvent ratio of 1:15 was selected.

### 4.4. Application of the Established Method to Polyphenol Profiling of Sea Buckthorn Juice Samples

The quantitative results obtained from the seven sea buckthorn juice samples demonstrate the applicability of the established HPLC-QqQ-MS/MS method to the determination of representative polyphenols in real samples. In addition to rutin, the method provided compound-specific concentration data for flavan-3-ols and phenolic acids, enabling the characterization of the measured polyphenol profiles.

**Figure 3:**
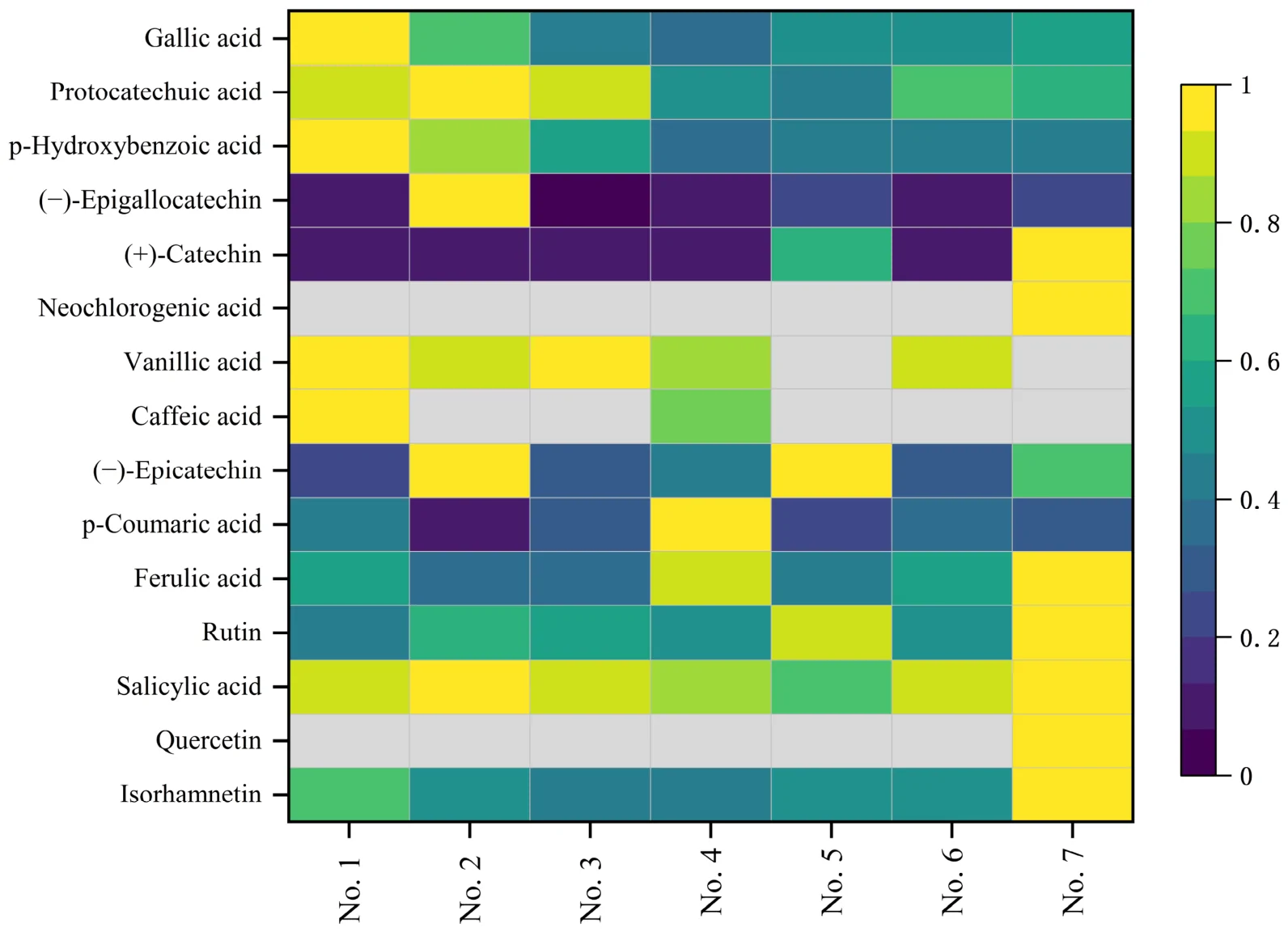
Heatmap showing the relative distribution patterns of 15 target polyphenols in seven sea buckthorn juice samples. Note: Samples Nos. 1–7 correspond to Daguo Shaji Nos. 1–7, respectively. For each compound, the mean concentration was normalized to the maximum mean concentration among the seven samples, with normalized values ranging from 0 to 1. Yellow and purple indicate higher and lower relative concentrations, respectively, while gray indicates compounds that were not detected (ND).

To further visualize these polyphenol profiles, a heatmap was used to illustrate the relative distribution patterns of the 15 target polyphenols across the seven sea buckthorn juice samples (Figure 3). Most target polyphenols, including rutin, salicylic acid, ferulic acid, and several phenolic acids and flavan-3-ols, were detected in all seven samples. Among these compounds, salicylic acid showed a relatively uniform distribution, whereas rutin showed higher relative concentrations in samples No. 5 and No. 7. Certain compounds displayed more pronounced relative concentration patterns in specific samples. For example, (−)-epigallocatechin exhibited a notably higher relative concentration in sample No. 2, (+)-catechin showed relatively high concentrations in samples Nos. 5 and 7, and (−)-epicatechin exhibited relatively high concentrations in samples Nos. 2 and 5, whereas p-coumaric acid showed its highest relative concentration in sample No. 4. In addition, some target polyphenols exhibited more restricted detection patterns, with neochlorogenic acid and quercetin detected only in sample No. 7, and caffeic acid detected only in samples Nos. 1 and 4. Overall, the heatmap provides a visual representation of the relative distribution and detection patterns of individual target polyphenols across the seven sea buckthorn juice samples, further demonstrating the applicability of the established method to the characterization of representative polyphenol profiles in actual sea buckthorn juice samples.

Beyond demonstrating the applicability of the established method, these polyphenol profiles may provide useful information for future investigations into the sensory and physicochemical characteristics of sea buckthorn juice. Previous studies have shown that different classes of polyphenols are associated with food properties such as astringency, bitterness, and oxidative stability [47,48,49]. In the present study, variations in the concentrations and relative contributions of individual polyphenols were observed among the seven sea buckthorn juice samples. These findings provide compositional information that may serve as a reference for future investigations into the relationships between polyphenol profiles and food quality characteristics.

The polyphenol profiles characterized in this study also provide a useful basis for further investigations into the functional properties of sea buckthorn. The present study primarily focused on the qualitative screening and quantitative analysis of individual polyphenols in different sea buckthorn samples, without further evaluating antioxidant activity or other functional and biological properties. Differences in polyphenol composition and content among samples reflect the diversity of their chemical profiles, whereas the specific relationships between these differences and biological activities remain to be verified through functional assessments. Future studies could build on the current qualitative and quantitative analyses of polyphenols by incorporating antioxidant activity assays, such as DPPH, ABTS, and FRAP, along with other relevant biological evaluations, to provide a more comprehensive understanding of the relationships between polyphenol composition and functional properties.

### 4.5. Analytical Characteristics of the Established Method and Comparison with Existing Methods

To further evaluate the analytical characteristics of the integrated analytical method, representative methods for polyphenol analysis in sea buckthorn were compared (Table 7). Conventional spectrophotometric assays are mainly used to estimate total phenolic content and therefore cannot readily distinguish or quantify individual polyphenols, whereas GC-MS is better suited to compounds amenable to gas-phase analysis, such as phenolic acids, and provides relatively limited coverage of highly polar and non-volatile polyphenols. HPLC-DAD is suitable for the routine quantification of major phenolic compounds, but its structural identification capability and selectivity are relatively limited, particularly when analyzing co-eluting compounds in complex matrices, which may affect the reliability of analytical results. Targeted LC-MS/MS enables the simultaneous quantification of multiple known polyphenols; however, its analytical coverage is inherently constrained by the predefined analyte panel. Meanwhile, the structural annotation and quantitative information obtained may also provide a basis for the further accumulation of sea buckthorn polyphenol data and the gradual improvement of chemical composition information in a bioactive compound database for specialty berries. In contrast, high-resolution QTOF-MS enables broad screening and structural annotation based on accurate mass measurements and fragmentation information, but its performance in routine targeted quantification is generally inferior to that of QqQ-MS/MS.

In this study, UPLC-QTOF-MS/MS was first employed for the broad screening and structural annotation of polyphenols in sea buckthorn juice, followed by the selection of 15 representative polyphenolic compounds for targeted quantification using HPLC-QqQ-MS/MS. First, high-resolution mass spectrometry provides accurate mass and fragment ion information, which helps distinguish co-eluting components in complex matrices and improves the reliability of structural annotation. Meanwhile, unlike targeted analytical methods that rely entirely on predefined analyte panels, the selection of target compounds in this study was based on the actual chemical composition information obtained by high-resolution mass spectrometry, providing a clearer basis for subsequent quantitative analysis. On this basis, the high sensitivity and selectivity of QqQ-MS/MS were utilized to compensate for the relative limitations of QTOF-MS in routine targeted quantification. Furthermore, compared with previous strategies combining QTOF-MS-based screening and structural annotation with DAD-based quantification, this study employed QqQ-MS/MS in multiple reaction monitoring (MRM) mode, using specific precursor-to-product ion transitions for target compound detection, thereby helping to reduce interference from co-eluting components in complex matrices and improve the selectivity and reliability of individual polyphenol quantification.

In this regard, the integrated analytical method can be applied to the investigation of samples whose polyphenol composition has not been fully characterized, providing a reference for compositional analysis and the development of targeted quantitative methods. In this study, the method was applied to seven sea buckthorn juice samples, providing information on the composition and concentration distribution of the target polyphenols in each sample and supporting comparisons of polyphenol composition among different samples and subsequent investigations of related compounds. Furthermore, the method allows the range of target compounds for quantification to be further expanded according to the chemical composition of actual samples and subsequent analytical needs, providing a reference for the accumulation of related polyphenol compositional data and the gradual improvement of chemical composition information in bioactive compound databases for specialty berries.

## 5. Conclusions

This study developed an integrated analytical method combining UPLC-QTOF-MS/MS-based qualitative screening with HPLC-QqQ-MS/MS-based targeted quantification for polyphenol analysis in sea buckthorn juice. A total of 42 candidate polyphenols were screened by UPLC-QTOF-MS/MS, providing qualitative information to guide the selection of 15 representative compounds for subsequent targeted quantification. Optimization showed that acetonitrile–0.1% formic acid in water was suitable as the mobile-phase system, while extraction with 70% ethanol followed by treatment with 0.01 mol/L HCl and a sample-to-solvent ratio of 1:15 provided both relatively high target-analyte extraction and broad detection coverage. The established HPLC-QqQ-MS/MS method demonstrated satisfactory linearity, sensitivity, accuracy, and repeatability under the validated conditions. The method was successfully applied to the determination of 15 target polyphenols in seven sea buckthorn juice samples, providing quantitative information for the characterization of their polyphenol composition. Overall, the integrated method links sample-informed high-resolution mass spectrometric screening and structural annotation with validated targeted quantification, providing a reliable analytical approach for polyphenol profiling and quantitative analysis in sea buckthorn juice.

## Figures and Tables

**Table 1:** Screening and annotation of polyphenolic compounds in sea buckthorn juice by UPLC-QTOF-MS/MS.

No.	RT (min)	Component Name	Molecular Formula	Observed *m*/*z*	Mass Error (ppm)	MS/MS Fragment Ions (*m*/*z*)
1	1.34	Gentisic acid	C_7_H_6_O_4_	153.0187	−4.4	109.0292
2	1.45	Gallic acid *	C_7_H_6_O_5_	169.0141	−0.7	125.0240
3	1.89	(−)-Epigallocatechin *	C_15_H_14_O_7_	305.0662	−1.4	219.0654, 125.0240
4	1.98	Protocatechuic acid *†	C_7_H_6_O_4_	153.0193	−0.5	—
5	2.03	Syringic acid †	C_9_H_10_O_5_	197.0451	−2.1	—
6	2.51	Procyanidin B3	C_30_H_26_O_12_	577.1354	0.4	425.0868, 407.0775, 289.0717, 245.0812
7	2.62	p-Hydroxybenzoic acid *	C_7_H_6_O_3_	137.0242	−1.6	93.0342
8	2.84	(+)-Catechin *	C_15_H_14_O_6_	289.0718	0.2	245.0818, 205.0506, 203.0712, 151.0394, 137.0244, 109.0293
9	2.87	Neochlorogenic acid *	C_16_H_18_O_9_	353.0867	−3.1	191.0556, 179.0347, 173.0450
10	2.91	p-Coumaric acid *	C_9_H_8_O_3_	163.0399	−1.2	119.0501
11	2.95	4-Hydroxy-3,5-dimethoxycinnamic acid	C_11_H_12_O_5_	223.0611	−0.5	208.0376, 193.0140, 179.0713, 164.0475, 149.0242
12	3.19	Quercetin-3-O-sophoroside-7-O-rhamnoside	C_33_H_40_O_21_	771.2000	1.4	447.0915, 301.0344, 271.0244
13	3.22	Caffeic acid *	C_9_H_8_O_4_	179.0347	−1.7	135.0446
14	3.24	Ferulic acid *	C_10_H_10_O_4_	193.0505	−0.5	134.0372
15	3.43	(−)-Epicatechin *	C_15_H_14_O_6_	289.0718	0.3	245.0822, 203.0711, 179.0340, 151.0396, 137.0241, 125.0241, 109.0293
16	3.44	Tellimagrandin I	C_34_H_26_O_22_	785.0869	3.3	125.0241
17	3.46	Isorhamnetin-3-O-rutinoside-7-O-glucoside	C_34_H_42_O_21_	785.2153	0.9	639.1574, 623.1623, 477.1040, 315.0507, 300.0276
18	3.58	Quercetin-3-O-rutinoside-7-O-glucoside	C_33_H_40_O_21_	771.1998	1.1	609.1472, 463.0874, 301.0338, 300.0275, 271.0249, 178.9982, 151.0035
19	3.85	Kaempferol-3-O-sophoroside-7-O-rhamnoside	C_33_H_40_O_20_	755.2047	0.9	609.1465, 431.1434, 255.0295
20	4.12	Vanillic acid *	C_8_H_8_O_4_	167.0348	−1.1	152.0109, 108.0214
21	4.17	Isorhamnetin-3-O-arabinoglucoside	C_27_H_30_O_16_	609.1466	0.8	477.1039, 255.0297, 151.0033
22	4.18	Kaempferol-3-O-rutinoside-7-O-rhamnoside	C_33_H_40_O_19_	739.2091	−0.1	284.0324, 227.0347
23	4.23	Coreopsin	C_21_H_22_O_10_	433.1136	−1.1	271.0605
24	4.38	Rutin *	C_27_H_30_O_16_	609.1468	1.1	301.0344, 300.0275, 271.0247, 255.0298, 178.9984, 151.0034
25	4.56	5-Hydroxy-6,7,3′,4′,5′-pentamethoxyflavone	C_20_H_20_O_8_	387.1079	−1.6	357.0980, 343.0460
26	4.57	Quercetin-7-O-glucoside	C_21_H_20_O_12_	463.0883	0.1	301.0341, 300.0275, 271.0247, 255.0298, 178.9984, 151.0035
27	4.85	Kaempferol-3-O-neohesperidoside	C_27_H_30_O_15_	593.1519	1.2	285.0401, 284.0324, 255.0297, 151.0039
28	4.97	Isorhamnetin-3-O-neohesperidoside	C_28_H_32_O_16_	623.1623	0.8	477.1038, 315.0507, 314.0431, 300.0271, 271.0247
29	5.03	Syringetin-3-O-rutinoside	C_29_H_34_O_17_	653.1729	0.9	345.0614, 330.0372
30	5.05	Quercetin-7-O-rhamnoside	C_21_H_20_O_11_	447.0934	0.3	255.0298, 151.0036
31	5.18	Isorhamnetin-3-O-glucoside	C_22_H_22_O_12_	477.1041	0.5	315.0487, 314.0432, 271.0246, 243.0296, 151.0036
32	5.19	Syringetin-3-O-glucoside	C_23_H_24_O_13_	507.1145	0.2	344.0535, 330.0374, 151.0036
33	5.26	Resveratrol	C_14_H_12_O_3_	227.0711	−1.0	185.0597, 143.0710
34	5.32	Aromadendrin	C_15_H_12_O_6_	287.0561	−0.1	177.0191, 151.0035, 135.0450
35	5.37	Salicylic acid *†	C_7_H_6_O_3_	137.0242	−1.5	—
36	5.44	Myricetin †	C_15_H_10_O_8_	317.0296	−2.1	—
37	5.49	Astragalin	C_21_H_20_O_11_	447.0933	0.1	285.0403, 257.0451, 227.0345
38	6.17	Kaempferol 3-O-α-L-[6‴-p-coumaroyl-β-D-glucopyranosyl-(1→2)-rhamnopyranoside]	C_36_H_36_O_17_	739.1876	−0.5	447.0929, 285.0399
39	6.50	Quercetin *	C_15_H_10_O_7_	301.0343	−3.7	151.0029
40	6.52	Kaempferol-7-O-α-L-rhamnoside	C_21_H_20_O_10_	431.0986	0.6	284.0326, 151.0033
41	6.71	Isorhamnetin-3-O-rhamnoside	C_22_H_22_O_11_	461.1087	−0.5	315.0503, 314.0423
42	7.75	Isorhamnetin *	C_16_H_12_O_7_	315.0512	0.6	300.0260, 151.0027

Note: *, compounds selected for HPLC-QqQ-MS/MS quantification; †, tentative annotations based on accurate-mass matching and database/literature evidence; —, no reliable diagnostic MS/MS fragment ions obtained.

**Table 2:** MRM parameters for the 15 target polyphenolic compounds.

No.	Compound	Precursor Ion (*m*/*z*)	Product Ions (*m*/*z*)	Fragmentor Voltage (V)	Collision Energies (eV)
1	Gallic acid	169.0	125.1/79.1	80	10/25
2	Protocatechuic acid	153.0	109.1/91.2	60	10/30
3	p-Hydroxybenzoic acid	137.0	92.9/65.0	60	10/30
4	(−)-Epigallocatechin	305.1	219.1/125.0	120	10/20
5	(+)-Catechin	288.9	244.9/202.9/108.9	130	10/15/25
6	Neochlorogenic acid	353.1	191.1/179.0	80	10/10
7	Vanillic acid	167.0	152.1/108.0	50	10/20
8	Caffeic acid	179.1	135.1/106.9	100	15/25
9	(−)-Epicatechin	289.0	245.0/203.0/108.9	125	10/15/30
10	p-Coumaric acid	163.0	119.1/93.0	70	15/35
11	Ferulic acid	193.1	177.9/134.0	70	10/15
12	Rutin	609.1	299.8/270.8	105	40/60
13	Salicylic acid	137.1	93.0/65.1	80	15/35
14	Quercetin	301.0	178.9/151.0	120	10/20
15	Isorhamnetin	315.1	300.0/151.0	100	20/25

Note: All target analytes were detected in negative ESI mode; product ions and the corresponding collision energies are listed in the same order.

**Table 3:** Effects of the extraction system and sample-to-solvent ratio on the extraction of target polyphenolic compounds.

Extraction System	Sum of 15 Target Polyphenols (μg/g)	Number of Detected Target Polyphenols
A. Extraction system (sample-to-solvent ratio fixed at 1:10, 2.00 g:20 mL)		
Methanol	2.31	6
Ethanol	3.03	8
Ethyl acetate	28.74	10
Water	25.12	13
0.01 mol/L HCl	24.67	13
70% methanol	16.03	11
70% ethanol	3.44	9
70% methanol–0.01 mol/L HCl	28.10	13
70% ethanol–0.01 mol/L HCl	30.75	13
Ethyl acetate–0.01 mol/L HCl	29.95	12
B. Sample-to-solvent ratio (extraction system fixed at 70% ethanol–0.01 mol/L HCl)		
1:5 (2.00 g:10 mL)	29.68	12
1:10 (2.00 g:20 mL)	31.62	13
1:15 (2.00 g:30 mL)	32.52	13
1:20 (2.00 g:40 mL)	31.95	13
1:25 (2.00 g:50 mL)	30.77	13

Note: The sum of 15 target polyphenols represents the sum of the quantified concentrations of the 15 target compounds.

**Table 4:** Calibration characteristics and sensitivity parameters for the 15 target polyphenolic compounds.

No.	Compound	Linear Range (ng/mL)	Regression Equation	R^2^	LOD (μg/g)	LOQ (μg/g)
1	Gallic acid	10–500	Y = 2505.0520X − 24,775.9111	0.9995	0.0028	0.0094
2	Protocatechuic acid	20–500	Y = 3894.0970X − 42,508.1841	0.9980	0.0037	0.0123
3	p-Hydroxybenzoic acid	10–500	Y = 4418.2574X − 11,288.0631	0.9990	0.0010	0.0034
4	(−)-Epigallocatechin	50–500	Y = 19.3217X − 34.2238	0.9996	0.0054	0.0179
5	(+)-Catechin	10–500	Y = 335.5616X − 2868.0011	0.9976	0.0010	0.0034
6	Neochlorogenic acid	10–500	Y = 341.5095X − 2986.0804	0.9998	0.0011	0.0035
7	Vanillic acid	10–500	Y = 130.0630X − 861.2879	0.9977	0.0020	0.0068
8	Caffeic acid	10–500	Y = 3176.7455X + 16,764.7442	0.9981	0.0022	0.0072
9	(−)-Epicatechin	10–500	Y = 396.5372X − 3669.5592	0.9998	0.0013	0.0043
10	p-Coumaric acid	10–500	Y = 9737.0611X + 9801.2376	0.9992	0.0010	0.0033
11	Ferulic acid	20–500	Y = 343.7020X − 1793.5353	0.9996	0.0015	0.0049
12	Rutin	10–500	Y = 213.4082X − 1136.2552	0.9994	0.0013	0.0042
13	Salicylic acid	20–500	Y = 7113.9216X − 79,608.0629	0.9974	0.0033	0.0109
14	Quercetin	10–500	Y = 361.8198X − 3182.3443	0.9994	0.0021	0.0071
15	Isorhamnetin	20–500	Y = 146.5425X − 2446.4223	0.9968	0.0032	0.0107

**Table 5:** Recoveries and RSDs of 15 target polyphenolic compounds at three spiking levels (*n* = 6).

No.	Compound	50 μg/kg		100 μg/kg		200 μg/kg	
		Recovery (%)	RSD (%)	Recovery (%)	RSD (%)	Recovery (%)	RSD (%)
1	Gallic acid	79.1	4.0	84.6	2.6	98.7	0.8
2	Protocatechuic acid	83.4	2.2	92.3	0.9	101.4	1.3
3	p-Hydroxybenzoic acid	80.3	3.1	87.7	1.7	92.8	2.0
4	(−)-Epigallocatechin	77.0	3.9	85.0	1.5	100.5	2.3
5	(+)-Catechin	86.3	4.4	94.2	3.7	103.2	3.5
6	Neochlorogenic acid	84.6	4.1	90.7	2.6	93.6	1.4
7	Vanillic acid	78.2	3.6	81.4	3.2	84.5	2.2
8	Caffeic acid	83.0	3.2	88.5	2.4	92.7	2.3
9	(−)-Epicatechin	80.8	2.4	88.4	2.5	84.5	3.7
10	p-Coumaric acid	90.8	3.3	94.2	2.6	81.7	1.1
11	Ferulic acid	81.2	3.3	99.0	4.2	101.7	2.9
12	Rutin	78.8	2.2	85.7	3.9	91.4	2.8
13	Salicylic acid	95.8	3.3	105.7	3.2	101.6	2.0
14	Quercetin	79.4	2.5	82.3	1.1	87.9	1.4
15	Isorhamnetin	76.8	3.1	85.5	2.1	89.2	2.4

Note: The values of 50, 100, and 200 μg/kg represent the spiking levels of the target analytes in the sample. For rutin, the original juice was diluted prior to standard addition to reduce its endogenous concentration, and the spiking levels of 50, 100, and 200 μg/kg, as well as the recovery evaluation, were based on the diluted juice samples.

**Table 6:** Sum of the 15 target polyphenols and rutin proportion in seven large-fruited sea buckthorn juice samples (*n* = 3).

Samples	Sum of 15 Target Polyphenols (μg/g)	Number of Detected Target Polyphenols	Rutin Content (μg/g)	Rutin Proportion (%)
Daguo Shaji No. 1	34.25 ± 0.09	13	30.05 ± 0.06	87.74
Daguo Shaji No. 2	60.18 ± 0.44	12	44.36 ± 0.58	73.72
Daguo Shaji No. 3	43.84 ± 1.41	12	40.75 ± 1.36	92.96
Daguo Shaji No. 4	41.38 ± 0.10	13	37.25 ± 0.06	90.01
Daguo Shaji No. 5	81.73 ± 0.79	11	66.09 ± 0.83	80.86
Daguo Shaji No. 6	40.16 ± 1.02	12	36.40 ± 1.09	90.63
Daguo Shaji No. 7	94.86 ± 1.74	13	72.10 ± 1.26	76.01

Note: Data are presented as mean ± standard deviation (*n* = 3); the sum of 15 target polyphenols represents the sum of the quantified concentrations of the 15 target compounds; rutin proportion was calculated as (mean rutin content/sum of the 15 target polyphenols) × 100%.

**Table 7:** Characteristics of different analytical methods applied to polyphenol analysis in sea buckthorn.

Method	Analytical Scope	Main Features and Limitations	Reference
UV-Vis	Total phenolic content	Rapid and low-cost, but unable to distinguish and quantify individual polyphenols.	[13]
GC-MS	Tentative identification of 17 phenolic acids	Suitable for phenolic acids and small molecules amenable to GC analysis, but limited for highly polar and non-volatile polyphenols.	[50]
HPLC-DAD	Simultaneous analysis of 9 phenolic acids	Suitable for routine quantification of known compounds, but limited in structural identification and selectivity in complex matrices.	[51]
LC-MS/MS	Predefined analytical scope based on 127 phenolic standards	High sensitivity and selectivity for targeted analysis, but coverage is limited by the predefined analyte panel.	[22]
QTOF-MS	44 chromatographic peaks detected, of which 33 were identified	Provides broad candidate screening and structural annotation, but is less suited than QqQ-MRM for routine targeted quantification.	[18]
QTOF + DAD	41 compounds tentatively identified and 12 phenolic compounds quantified, mainly flavonols	Combines high-resolution structural annotation with optical quantification, but quantitative selectivity in complex matrices is relatively limited.	[52]
QTOF + QqQ	42 candidate polyphenols screened; 15 representative compounds targeted for quantification, covering phenolic acids, flavan-3-ols, and flavonols	Combines broad screening with MRM-targeted quantification, improving selectivity for target compounds in complex matrices.	This study

## Data Availability

The original contributions presented in this study are included in the article/Supplementary Materials. Further inquiries can be directed to the corresponding authors.

## Supplementary Materials

The following supporting information can be downloaded at https://www.mdpi.com/article/10.3390/foods15213773/s1, Figure S1. Extracted ion chromatograms of 15 target polyphenolic compounds in sea buckthorn juice extract; Table S1. Contents of individual target polyphenols in sea buckthorn juice obtained using different extraction systems (μg/g); Table S2. Contents of individual target polyphenols in sea buckthorn juice obtained at different sample-to-solvent ratios (μg/g); Table S3. Contents of the 15 target polyphenols in seven large-fruited sea buckthorn juice samples (μg/g).
