# Supplementary material for: Development of an Integrated UPLC-QTOF-MS/MS and HPLC-QqQ-MS/MS Method for Polyphenol Analysis in Sea Buckthorn Juice
Source: Foods. 2026 Oct 22;15(21):3773. doi: 10.3390/foods15213773 (PMC13763006; doi:10.3390/foods15213773)
Supplement: Supplementary file 1 [file foods-15-03773-s001.zip › foods-4599439-supplementary.pdf]

Supporting Information

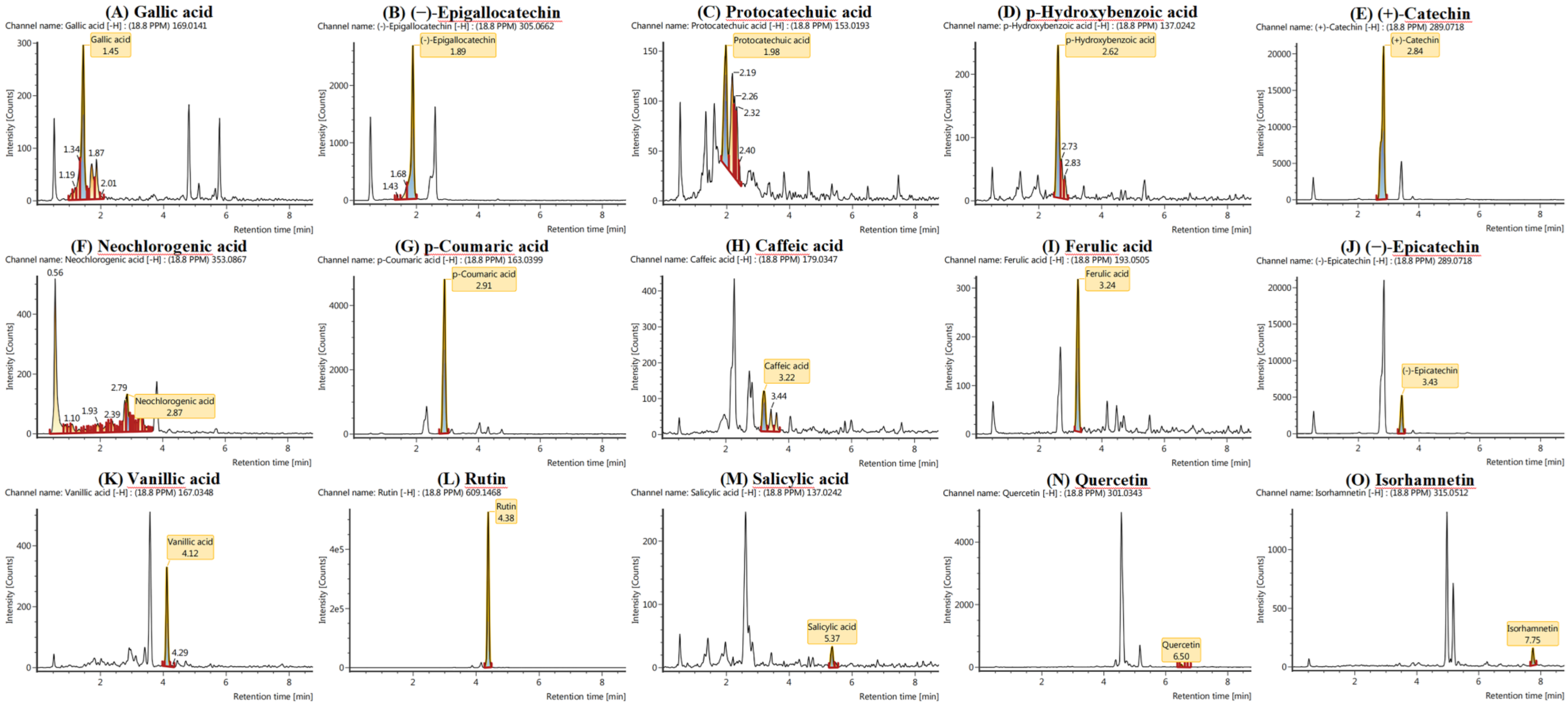

Figure S1. Extracted ion chromatograms of 15 target polyphenolic compounds in sea buckthorn juice extract.

Note: (A) Gallic acid; (B) (−)-Epigallocatechin; (C) Protocatechuic acid; (D) p-hydroxybenzoic acid; (E) (+)-Catechin; (F) Neochlorogenic acid; (G) p-coumaric acid; (H) Caffeic acid; (I) Ferulic acid; (J) (−)-Epicatechin; (K) Vanillic acid; (L) Rutin; (M) Salicylic acid; (N) Quercetin; and (O) Isorhamnetin.

Table S1. Contents of individual target polyphenols in sea buckthorn juice obtained using different extraction systems (μg/g)

Target compound	Methanol	Ethanol	Ethyl acetate	Ultrapure water	0.01 mol/L HCl	70% methanol	70% ethanol	70% methanol–0.01 mol/L HCl	70% ethanol–0.01 mol/L HCl	Ethyl acetate–0.01 mol/L HCl
Gallic acid	ND	0.017	0.046	0.128	0.147	0.022	0.016	0.121	0.142	0.122
Protocatechuic acid	0.029	0.032	0.074	0.185	0.161	0.045	0.033	0.184	0.212	0.252
p-Hydroxybenzoic acid	0.020	0.019	0.035	0.076	0.076	0.058	0.028	0.087	0.086	0.101
(−)-Epigallocatechin	ND	ND	0.857	0.397	0.419	ND	ND	0.995	1.04	0.404
(+)-Catechin	0.421	0.745	3.25	1.79	1.04	1.05	0.581	1.80	1.83	1.27
Neochlorogenic acid	ND	ND	ND	ND	ND	ND	ND	ND	ND	ND
Vanillic acid	ND	0.014	0.015	0.021	0.031	0.029	0.015	0.030	0.035	0.034
Caffeic acid	ND	ND	0.023	0.024	0.024	0.024	0.020	0.023	0.022	0.025
(−)-Epicatechin	0.042	0.078	0.413	0.106	0.074	0.115	0.080	0.120	0.116	0.092
p-Coumaric acid	0.019	0.019	0.036	0.135	0.101	0.076	0.032	0.078	0.084	0.133
Ferulic acid	ND	ND	ND	0.026	0.023	0.018	ND	0.019	0.028	0.020
Rutin	1.78	2.11	23.99	22.19	22.53	14.57	2.63	24.59	27.10	27.49
Salicylic acid	ND	ND	ND	0.021	0.026	0.012	ND	0.032	0.030	0.018
Quercetin	ND	ND	ND	ND	ND	ND	ND	ND	ND	ND
Isorhamnetin	ND	ND	ND	0.017	0.019	ND	ND	0.018	0.020	ND

Note: The sample-to-solvent ratio was 1:10 (2.00 g: 20 mL); ND, not detected.

Table S2. Contents of individual target polyphenols in sea buckthorn juice obtained at different sample-to-solvent ratios (μg/g)

Target compound	1:5 (2.00 g: 10 mL)	1:10 (2.00 g: 20 mL)	1:15 (2.00 g: 30 mL)	1:20 (2.00 g: 40 mL)	1:25 (2.00 g: 50 mL)
Gallic acid	0.144	0.155	0.183	0.173	0.172
Protocatechuic acid	0.190	0.214	0.217	0.237	0.239
p-Hydroxybenzoic acid	0.071	0.070	0.072	0.073	0.072
(−)-Epigallocatechin	ND	0.978	1.16	1.02	1.08
(+)-Catechin	1.60	1.85	1.95	1.89	1.89
Neochlorogenic acid	ND	ND	ND	ND	ND
Vanillic acid	0.040	0.034	0.044	0.038	0.031
Caffeic acid	0.028	0.024	0.026	0.025	0.027
(−)-Epicatechin	0.105	0.122	0.144	0.128	0.128
p-Coumaric acid	0.085	0.095	0.088	0.091	0.101
Ferulic acid	0.033	0.029	0.025	0.030	0.025
Rutin	27.34	28.00	28.58	28.20	26.95
Salicylic acid	0.025	0.024	0.025	0.025	0.036
Quercetin	ND	ND	ND	ND	ND
Isorhamnetin	0.021	0.021	0.022	0.023	0.023

Note: The extraction solvent was 70% ethanol–0.01 mol/L HCl; ND, not detected.

Table S3. Contents of the 15 target polyphenols in seven large-fruited sea buckthorn juice samples (μg/g)

Target compound	Daguo Shaji No. 1	Daguo Shaji No. 2	Daguo Shaji No. 3	Daguo Shaji No. 4	Daguo Shaji No. 5	Daguo Shaji No. 6	Daguo Shaji No. 7
Gallic acid	0.177 ± 0.004^{ab}	0.1193 ± 0.0007^{c}	0.082 ± 0.001^{d}	0.069 ± 0.003^{e}	0.091 ± 0.001^{d}	0.092 ± 0.004^{d}	0.099 ± 0.002^{c}
Protocatechuic acid	0.234 ± 0.002^{a}	0.255 ± 0.004^{a}	0.225 ± 0.003^{a}	0.1315 ± 0.0006^{b}	0.117 ± 0.001^{b}	0.185 ± 0.007^{c}	0.156 ± 0.006^{d}
p-Hydroxybenzoic acid	0.081 ± 0.003^{a}	0.065 ± 0.002^{b}	0.0451 ± 0.0008^{c}	0.031 ± 0.001^{d}	0.036 ± 0.001^{d}	0.0347 ± 0.0006^{d}	0.033 ± 0.001^{d}
(−)-Epigallocatechin	1.29 ± 0.02^{b}	12.27 ± 0.22^{a}	0.741 ± 0.033^{c}	1.25 ± 0.04^{b}	3.06 ± 0.09^{b}	1.25 ± 0.05^{b}	2.47 ± 0.10^{b}
(+)-Catechin	2.03 ± 0.02^{c}	2.28 ± 0.06^{c}	1.63 ± 0.03^{c}	1.98 ± 0.07^{c}	11.54 ± 0.10^{b}	1.80 ± 0.03^{c}	19.18 ± 0.45^{a}
Neochlorogenic acid	ND	ND	ND	ND	ND	ND	0.126 ± 0.004
Vanillic acid	0.043 ± 0.001^{a}	0.039 ± 0.002^{ab}	0.041 ± 0.001^{ab}	0.037 ± 0.001^{b}	ND	0.0384 ± 0.0008^{ab}	ND
Caffeic acid	0.0280 ± 0.0009^{a}	ND	ND	0.0211 ± 0.0005^{b}	ND	ND	ND
(−)-Epicatechin	0.153 ± 0.006^{c}	0.705 ± 0.023^{a}	0.189 ± 0.003^{c}	0.313 ± 0.010^{b}	0.691 ± 0.028^{a}	0.216 ± 0.006^{c}	0.504 ± 0.004^{b}
p-Coumaric acid	0.093 ± 0.004^{a}	0.0240 ± 0.0008^{d}	0.0716 ± 0.0007^{b}	0.226 ± 0.005^{a}	0.0482 ± 0.0008^{c}	0.076 ± 0.001^{b}	0.063 ± 0.002^{b}
Ferulic acid	0.0213 ± 0.0009^{c}	0.0140 ± 0.0002^{d}	0.0138 ± 0.0005^{d}	0.0344 ± 0.0008^{b}	0.0168 ± 0.0005^{c}	0.0232 ± 0.0007^{c}	0.0391 ± 0.0002^{a}
Rutin	30.05 ± 0.06^{d}	44.36 ± 0.58^{c}	40.75 ± 1.36^{c}	37.25 ± 0.06^{c}	66.09 ± 0.83^{b}	36.40 ± 1.09^{c}	72.10 ± 1.26^{a}
Salicylic acid	0.0294 ± 0.0009^{ab}	0.0320 ± 0.0003^{a}	0.0295 ± 0.0009^{ab}	0.0286 ± 0.0003^{ab}	0.0227 ± 0.0006^{c}	0.0305 ± 0.0009^{ab}	0.0337 ± 0.0007^{a}
Quercetin	ND	ND	ND	ND	ND	ND	0.025 ± 0.001
Isorhamnetin	0.0220 ± 0.0002^{b}	0.0147 ± 0.0003^{d}	0.0137 ± 0.0002^{d}	0.0135 ± 0.0002^{d}	0.0157 ± 0.0007^{c}	0.0153 ± 0.0005^{cd}	0.030 ± 0.001^{a}

Note: Data are presented as mean ± standard deviation ($n = 3$); ND, not detected; different lowercase superscript letters indicate significant differences among samples ($p < 0.05$).
